# Oncolytic Vaccinia Virus Harboring *Aphrocallistes vastus* Lectin Inhibits the Growth of Hepatocellular Carcinoma Cells

**DOI:** 10.3390/md20060378

**Published:** 2022-06-04

**Authors:** Riqing Jiang, Yufeng Qiu, Xiaomei Zhang, Ningning Zhou, Xiaoyuan Jia, Kan Chen, Yanrong Zhou, Ting Ye, Gongchu Li

**Affiliations:** College of Life Sciences and Medicine, Zhejiang Sci-Tech University, Hangzhou 310018, China; jiangrq@mails.zstu.edu.cn (R.J.); yfqiu@mails.zstu.edu.cn (Y.Q.); zhangxm@mails.zstu.edu.cn (X.Z.); znnzjlg@mails.zstu.edu.cn (N.Z.); xyjia@zstu.edu.cn (X.J.); chenkan@zstu.edu.cn (K.C.); zhouyanrong@zstu.edu.cn (Y.Z.)

**Keywords:** oncolytic vaccinia virus, viral replication, *Aphrocallistes vastus* lectin, Huh7 cells

## Abstract

Oncolytic vaccinia virus has been developed as a novel cancer therapeutic drug in recent years. Our previous studies demonstrated that the antitumor effect of oncolytic vaccina virus harboring Aphrocallistes vastus lectin (oncoVV-AVL) was significantly enhanced in several cancer cells. In the present study, we investigated the underlying mechanisms of AVL that affect virus replication and promote the antitumor efficacy of oncolytic virus in hepatocellular carcinoma (HCC). Our results showed that oncoVV-AVL markedly exhibited antitumor effects in both hepatocellular carcinoma cell lines and a xenograft mouse model. Further investigation illustrated that oncoVV-AVL could activate tumor immunity by upregulating the expression of type I interferons and enhance virus replication by inhibiting ISRE mediated viral defense response. In addition, we inferred that AVL promoted the ability of virus replication by regulating the PI3K/Akt, MAPK/ERK, and Hippo/MST pathways through cross-talk Raf-1, as well as metabolism-related pathways. These findings provide a novel perspective for the exploitation of marine lectins in oncolytic therapy.

## 1. Introduction

Oncolytic viruses are a class of natural or recombinant viruses that selectively infect and replicate in cancer cells, resulting in cell lysis while sparing normal tissue [1]. Currently, many oncolytic virus vectors are widely used in preclinical studies or clinical trials, including herpes simplex virus, adenovirus, Newcastle disease virus, poliovirus, vaccinia virus, reovirus, and parvovirus [2].

As a kind of double-stranded DNA viruses, vaccinia viruses have been used as oncolytic agents because of their inherent biological properties that distinguish them from other oncolytic viruses. Humans have an understanding about the biological characteristics and pathogenesis of vaccinia virus resulting from the eradication of smallpox. Vaccinia virus is easily genetically modified and shows good genetic stability and tropism for tumor cells [3,4,5]. In recent years, many genetically modified oncolytic vaccinia viruses have been assessed in clinical trials [6]. JX-594, a famous thymidine kinase (TK)-deleted oncolytic vaccinia virus with the insertion of human granulocyte-macrophage colony-stimulating factor/β-galactosidase, enhances oncolytic activity and was combined with sorafenib for the treatment of hepatocellular carcinoma in phase III clinical trials [7]. The TK-deleted vaccinia virus GLV-1h68 (GL-ONC1) harboring β-galactosidase and β-glucuronidase genes has also undergone clinical trials and showed good efficacy in head and neck squamous-cell carcinoma [8]. Moreover, recombinant vaccinia virus TG6002 with deletions of two genes (TK and ribonucleotide) and insertion of the suicide gene FCU1, is currently being evaluated in clinical trials [9]. In short, oncolytic vaccinia viruses have shown considerable safety and efficacy in clinical trials.

Lectins, a group of nonenzymatic, nonantibody, glycosylated, sugar-binding proteins, have been widely used in cancer diagnosis treatment and prognosis in combination with other biological methods [10,11]. Recently, the functions of marine lectins have been explored based on their characteristics, such as apoptosis elicitation, autophagy induction, and inhibition of angiogenesis [12,13,14,15]. Quite a few marine lectins have shown antitumor function through inducing apoptosis via several signaling pathways. For example, Fujji et al. reported that *Ibacus novemdentatus* lectin (iNol) demonstrated antiproliferative activity in Hela (ovarian), Caco-2 (colonic), MCF-7, and T47D (breast) cell lines. iNol induced Hela cells to apoptosis through DNA fragmentation and the intrinsic apoptosis pathway [16]. Terada et al. showed that *Mytilus galloprovincialis* lectin (MytiLec) could bind to the disaccharide melibiose on the surface of various cancer cells, such as Burkitt lymphoma, resulting in cytotoxicity to the cells [17]. *Crenomytilus grayanus* lectin (CGL) enhanced G2/M cell cycle arrest and induced Burkitt’s lymphoma cell apoptosis by interacting with the cellular surface glycan GB3 and the cleavage of poly ADP-ribose polymerase [18]. In addition, *Tachypleus tridentatus* lectin (TTL) [19], *Haliotis discus discus* sialic acid binding lectin (HddSBL) [20], *Dicentrarchus labrax* fucose-binding lectin (DLFBL) [21], *Strongylocentrotus purpuratus* rhamnose-binding lectin (SpRBL) [21], as well as White-spotted charr lectin (WCL) [22] were inserted into oncolytic virus and displayed cytotoxic effects against multiple cancer cells through inducing apoptosis. These findings show that marine lectins are an important candidate for tumor therapy.

We have previously demonstrated that oncolytic vaccinia virus harboring *Aphrocallistes vastus* lectin (oncoVV-AVL) had substantial antitumor effects on colorectal cancer, hepatocellular carcinoma, and cervical cancer [23,24]. However, the mechanism of oncolysis remains unclear. Here, we examined the oncolytic efficiency of oncoVV-AVL in hepatocellular carcinoma (HCC) cells and a xenograft mouse model, and explored the underlying molecular mechanism of oncoVV-AVL influencing viral replication in HCC.

## 2. Results

### 2.1. Cytotoxicity of OncoVV-AVL on HCC Cells

**Figure 1:**
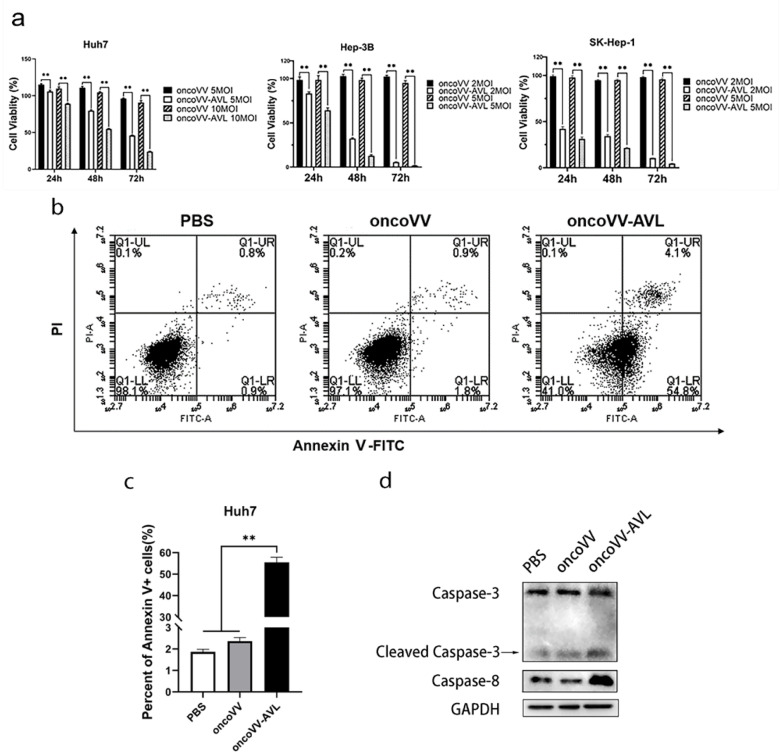
OncoVV-AVL promotes cell apoptosis in HCC cells. (**a**) Cell viability was observed by MTT assay in Huh7, Hep-3B, and SK-Hep-1 cells infected with oncoVV or oncoVV-AVL (** *p* < 0.01). (**b**) Apoptosis rate of Huh7 cells was detected by flow cytometry. Cells were treated with oncoVV or oncoVV-AVL (MOI 5) for 36 h. (**c**) Statistical results of apoptosis rate (** *p* < 0.01). (**d**) Expression of apoptosis-related protein was verified by Western blot, and compared with control PBS, with GAPDH serving as reference protein.

To examine the cytotoxicity of oncoVV-AVL on HCC cells, we used an MTT colorimetry assay to detect the cell viability of Huh7, Hep-3B, and SK-Hep-1 cells at 24, 48, and 72 h post infection with oncoVV or oncoVV-AVL. In Huh7, Hep-3B, and SK-Hep-1 cells, the cell viability during different periods was significantly lower in the oncoVV-AVL group compared with oncoVV-treated cells. The viability of the oncoVV-AVL-infected HCC cells significantly decreased with the time of infection, while that of the oncoVV-treated cells slightly decreased (Figure 1a), suggesting that oncoVV-AVL had a good antiproliferative effect on HCC cells.

To understand the inhibition mechanism of oncoVV-AVL in HCC cells, apoptosis was monitored in Huh7 cells for 36 h post-infection. The proportion of apoptotic cells in the oncoVV-AVL-treated group was 30-fold higher than in the PBS control (Figure 1b,c), implying that oncoVV-AVL promoted apoptosis in HCC cells. The result showed that caspase-8 and cleaved-caspase-3 in the oncoVV-AVL-treated group were also upregulated compared with the oncoVV-treated group (Figure 1d), indicating that oncoVV-AVL could trigger the apoptosis pathway in Huh7 cells.

### 2.2. Replication Ability of OncoVV-AVL in HCC Cells

**Figure 2:**
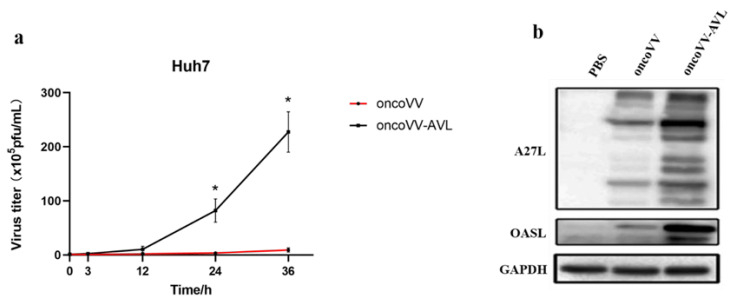
Promotion by AVL of viral replication in Huh7 cells. (**a**) Replication of oncoVV-AVL and oncoVV in Huh7 cells was investigated by TCID50 assay (* *p* < 0.05). (**b**) Expressions of A27L and OASL were detected through Western blotting.

TCID_50_ was used to evaluate the virus titer in cells infected with oncoVV and oncoVV-AVL, separately. Compared with oncoVV, oncoVV-AVL had significantly higher virus yields (Figure 2a). A27L is a protein located on the surface of intracellular viruses [25]. Ghosh et al. found 2′-5′-oligoadenylate synthetases-like protein (OASL) had opposite action on the replication of DNA and RNA viruses [26]. OASL enhanced RNA-sensor RIG-I-mediated antiviral response and inhibited the replication of RNA virus, while it inhibited cGAS activity to promote replication of DNA virus [26]. To further confirm the replication ability of oncoVV-AVL and the variation in OASL after infection of oncoVV-AVL, we tested the expressions of A27L and OASL, the results showed that both A27L and OASL expressions were obviously upregulated. We found that oncoVV-AVL may improve viral replication ability in Huh7 cells through upregulating the expression of OASL protein.

### 2.3. OncoVV-AVL Promotes Transcription of Type I Interferon in HCC Cells

**Figure 3:**
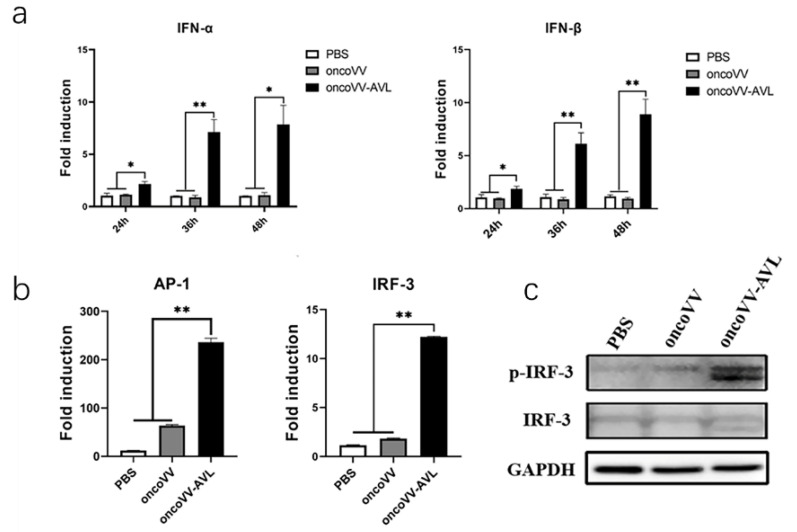
Upregulation of typeⅠinterferon transcription. (**a**) Evaluation of IFN-α/β transcription at 48 h post-infection with oncoVV or oncoVV-AVL in Huh7 cells. Comparative Ct (cycle threshold) value was used to investigate mRNA expression (* *p <* 0.05, ** *p <* 0.01). (**b**) Transcription activity of IRF-3 and AP-1. In dual-luciferase reporter gene assay, transfection efficiency was normalized by PRL-TK luciferase plasmid. Data are expressed as the mean ± SEM from at least three experimental repetitions (** *p <* 0.01). (**c**) Expressions of IRF-3 and p-IRF-3 determined by Western blotting.

Interferons (IFNs) are glycoproteins produced by cells in response to virus stimulation and promote antineoplastic immune responses in cancer cells [27]. To investigate the expression of interferons influenced by oncoVV-AVL, Huh7 cells were infected by oncoVV and oncoVV-AVL for 24, 36, or 48 h, and the expressions of type I IFNs (IFN-α, IFN-β) were detected. The results showed that both IFN-α and IFN-β expressions were enhanced via the infection of oncoVV-AVL, especially at 36 and 48 h post-infection (Figure 3a), indicating oncoVV-AVL could efficiently induce type I IFN production.

The transcription of IFN-β depends on the complex formed by phosphorylated IFN regulatory factor 3 (IRF3), and transcription factors AP-1 and NF-κB [28,29,30]. To elucidate the molecular mechanism of oncoVV-AVL elevating the expression of type I interferon, dual-luciferase reporter gene assays were performed to investigate the transcription activity of IRF-3 and AP-1. Compared with control PBS and oncoVV infection, the transcription activities of IRF-3 and AP-1 in oncoVV-AVL group were significantly upregulated (Figure 3b). Western blotting showed that the phosphorylation of IRF-3 was increased in oncoVV-AVL-infected cells (Figure 3c), suggesting that oncoVV-AVL could activate IRF-3 to increase IFN production.

### 2.4. Regulation of Antiviral Factors by OncoVV-AVL in HCC Cells

**Figure 4:**
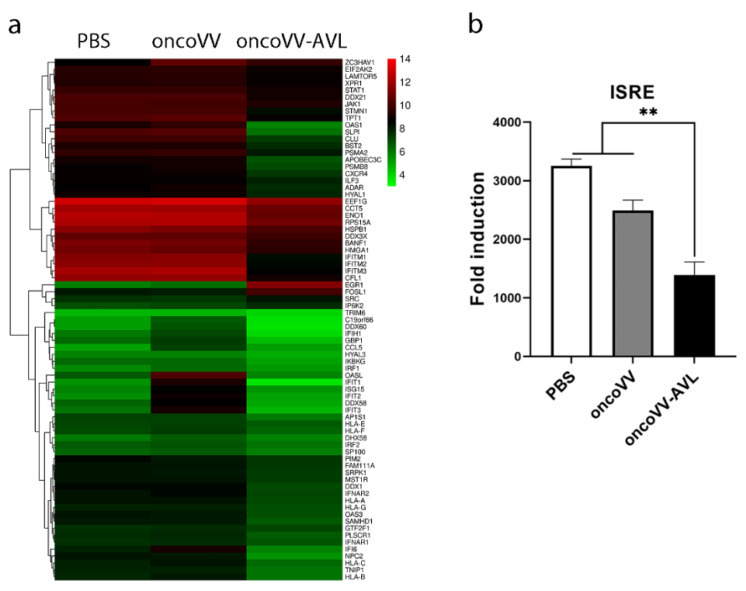
Transcriptomic verification of oncoVV-AVL. (**a**) Heatmap of genes in Huh7 cells. Transcriptomic sequencing was performed at 36 h post-infection with oncoVV and oncoVV-AVL (MOI 5), separately. The key includes a histogram of the distribution of log2 fold variation values for specific genes. (**b**) ISRE transactivation determined using dual-luciferase reporter assay (** *p <* 0.01).

To obtain an overview of the cell genes expressed in response to oncoVV-AVL infection, transcriptomic sequencing was performed. OmicStudio tools were used to compare the significance of the observed changes in gene expression among the three different groups. The results showed that many host defense factors were differentially downregulated (Figure 4a). The expressions of the antiviral factors of the oncoVV-AVL group, such as interferon-induced transmembrane proteins (IFITs), interferon-stimulated gene 15 (ISG15), 2′-5′-oligoadenylate synthetase (OAS), interleukin enhancer binding factor 3, phospholipid scramblase 1 (PLSCR1), and TNIP interacting protein 1 (TNIP1), were downregulated by more than two-fold compared with the oncoVV group at 36 h post-infection. Among them, OAS1, SLPI, IFITM3 and IFIT1 expressions were significantly downregulated by more than ten-fold, and IFIT1 expression decreased by 50 times. These data indicated that AVL could suppress the host’s antiviral response in Huh7 cells to facilitate viral replication.

IFNs initiate the transcription of IFN-stimulated genes (ISGs) through the JAK/STAT signaling pathway [31]. IFN-α and IFN-β bind to receptors IFNAR1 and IFNAR2, respectively [32], driving the activation of JAK and phosphorylation of STAT1 and STAT2 [33]. The ISGF3 complex, formed by phosphorylated STAT1, STAT2, and IRF9, binds to interferon-stimulated response elements (ISREs), enabling the expression of ISGs [34]. The results of the dual-luciferase reporter gene assay demonstrated that oncoVV-AVL significantly reduced the expression of ISRE (Figure 4b), suggesting that although oncoVV-AVL enhanced the production of IFN, the infection with oncoVV-AVL also downregulated the expression of ISGs through downregulating the level of ISRE in Huh7 cells.

### 2.5. Pathways Associated with Replication Ability of OncoVV-AVL

**Figure 5:**
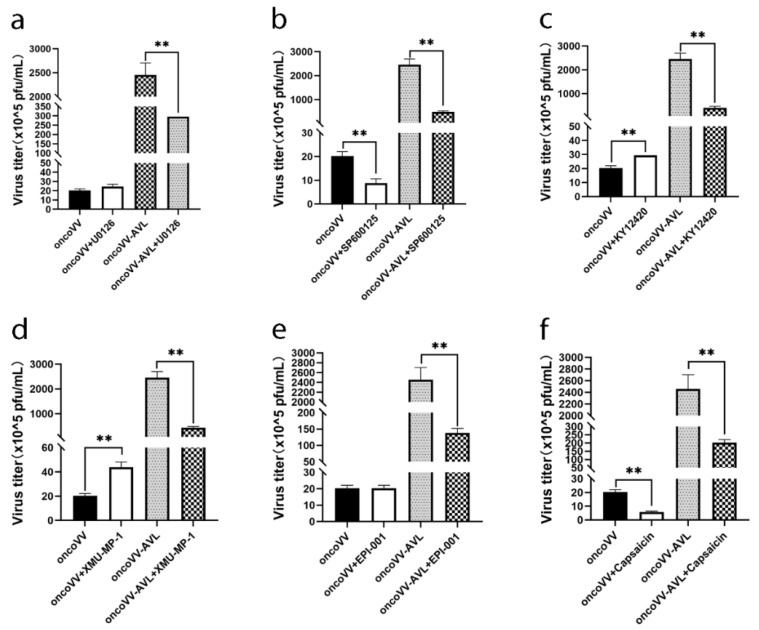
Virus yields in presence of activator or inhibitor. Huh7 cells were infected with oncoVV or oncoVV-AVL in presence of U0126 (**a**), SP600125 (**b**), KY12420 (**c**), XMU-MP-1 (**d**), EPI-001 (**e**), or capsaicin (**f**) (** *p* < 0.01).

To further explore which cell signaling pathway was affected by the infection of oncoVV-AVL, several inhibitors and activators of signaling pathways were used to assess the virus yields. Extracellular signal-regulated kinase (ERK) inhibitor U0126 significantly inhibited the replication of oncoVV-AVL, while C-Jun N-terminal kinase (JNK) inhibitor SP600125 inhibited the replication of both oncoVV and oncoVV-AVL in Huh7 cells (Figure 5a,b), indicating that AVL could interfere with ERK signaling. Phosphatidylinositol-3 family kinases (PI3K) regulate various cell processes including host defense. KY12420, a PI3K inhibitor, restrained the replication of oncoVV-AVL compared with the control (Figure 5c), indicating that AVL could improve oncoVV replication by activating PI3K. Moreover, mammalian sterile 20-like kinase 1/2 (MST1/2), as a critical kinase in the Hippo signaling pathway, enhances viral replication through blocking virus-induced TBK1/IKKε activity [35]. XMU-MP-1(an MST1/2 inhibitor) resisted the replication of oncoVV-AVL (Figure 5d), indicating that oncoVV-AVL promoted viral replication through the activation of MST1/2. In short, different kinases and phosphatases, such as PI3K, ERK, and MST1, serve as critical regulators in the replication of oncoVV-AVL in Huh7 cells.

Furthermore, we found that EPI-001, which modulated peroxisome proliferator activated receptor-gamma (PPARγ) as well as suppressed androgen receptor (AR) activity [36], significantly inhibited the replication of oncoVV-AVL compared with oncoVV (Figure 5f). Capsaicin, an agonist targeting the transient receptor potential vanilloid 1 (TRPV1), also showed a remarkable inhibiting effect on the replication of oncoVV and oncoVV-AVL. These findings showed that multiple signaling pathways influence the replication of oncoVV and oncoVV-AVL, and additional details need to be further elucidated.

### 2.6. OncoVV-AVL Suppressed HCC Growth In Vivo

**Figure 6:**
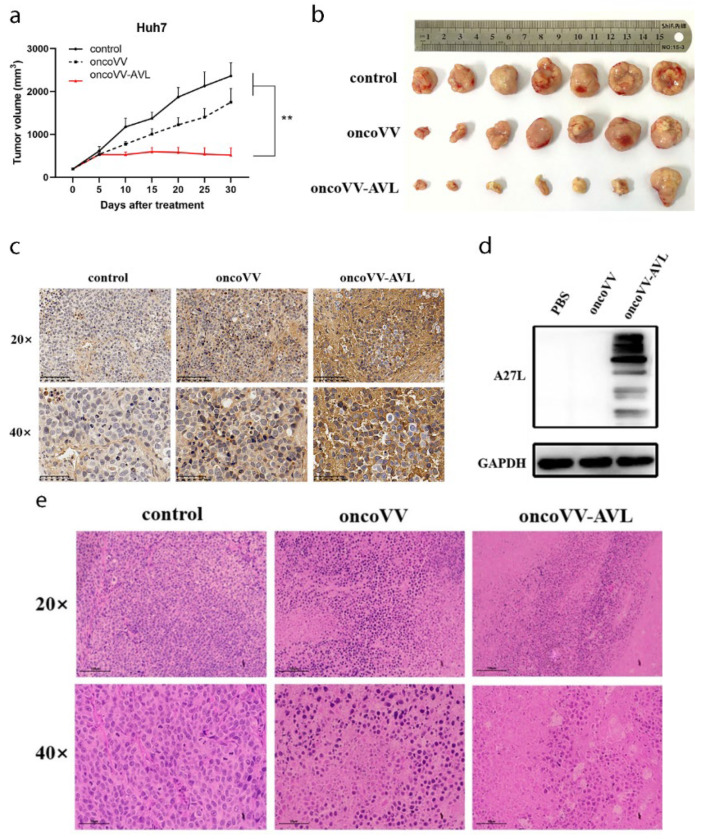
OncoVV-AVL suppressed tumor in vivo. (**a**) Volume growth curve of Huh7 tumors in Balb/c nude mice that were intratumorally injected with control 0.9% normal saline, oncoVV, or oncoVV-AVL (** *p* < 0.01). (**b**) Photographs of tumors from sacrificed mice. (**c**) Immunohistochemistry results of A27L on Huh7 tumors. (**d**) Expression of A27L determined by Western blotting. (**e**) H&E-staining result of mouse tumor tissue.

The mouse subcutaneous tumorigenesis model was established to confirm the anticancer effect of oncoVV-AVL in vivo. As shown by the tumor growth curves, compared with control PBS and oncoVV treatment, the oncoVV-AVL group showed robust antitumor effects in vivo (Figure 6a). On the 30th day post virus injection, tumors were removed from sacrificed mice, and the results showed that oncoVV-AVL significantly inhibited tumor growth (Figure 6b). In the immunohistochemistry assay, the darker brown in the cytoplasm implied the higher expression of A27L, suggesting a large number of viruses replicated in the oncoVV-AVL treatment (Figure 6c). Distinctly higher expression of A27L was observed in the oncoVV-AVL treatment through Western blot; in contrast, A27L was too low to detect in the oncoVV group (Figure 6d), which indicated that oncoVV-AVL could continuously replicate and was hard to clear by the host. In the hematoxylin and eosin (H&E)-stained assay, the nuclei were blue-purple, and the cytoplasm was red. Compared with the PBS and oncoVV groups, most tumor cells in the oncoVV-AVL group morphologically changed, with significantly more broken nuclei and intercellular substances, indicating there were obvious karyorrhexis and coagulative necrosis in the oncoVV-AVL group (Figure 6e) [37,38]. Taken together, these results indicated that arming with AVL can efficiently enhance the antitumor ability of oncoVV.

## 3. Discussion

For many years, it has been thought that the efficacy of oncolytic virotherapy is mediated through the lytic effect of the direct infection of cancer cells or the viral elicitation of antitumor immune responses [39]. In the current study, we demonstrated the oncolytic efficiency of oncoVV-AVL against HCC cell in vitro and in vivo. The results showed that oncoVV-AVL exhibited an efficient antitumor effect with a unique immunomodulatory mechanism, that is, inducing the production of type I IFNs while suppressing the ISRE-mediated viral defense response to promote self-replication. Our results support the idea that arming vaccinia virus with marine lectin can not only enhance the persistence of viral replication but also promote therapeutic efficacy [22].

Oncolytic viruses are an important branch of study in the tumor immunological therapy field. The key problem to be solved is how to activate the antitumor effect of the virus and prevent it from being cleared by the host defense system. During virus infection, the cells elicit a series of defense responses to protect themselves. Type I IFNs play a pivotal role in antiviral responses through inducing hundreds of ISGs. Among the numerous ISGs, ubiquitin-like protein ISG15, one of the most strongly and rapidly stimulated genes, can modulate the JAK/STAT signal pathway and directly inhibit viral replication [40]. In this study, the transcriptomic results showed that oncoVV-AVL significantly downregulated the transcription of ISG15, indicating oncoVV harboring AVL suppressed the antiviral response of the cancer cells to promote viral replication. OASL is another well-characterized member of the ISGs. Ghosh et al. reported that OASLs have opposite effects on the replication of RNA and DNA viruses, and can increase the replication of DNA virus [26]. Consistently, our results revealed that oncoVV-AVL upregulated the expression of OASL, suggesting oncoVV-AVL promoted vaccinia virus replication. Together with the findings that multiple antiviral factors such as AM111A, SRPK1, IFI16, and IFIT family member [41,42,43] are downregulated by oncoVV-AVL, we propose that although oncoVV-AVL enhances the production of IFNs, it can still downregulate the production of ISGs and replicate more efficiently against tumors.

Viral replication, a critical problem in oncolytic therapy, is a complex process and completely dependent on various cellular pathways. The virus usually regulates intracellular signaling pathways for persistent replication through direct or indirect control of phosphatases and kinases. For instance, the PI3K/Akt pathway is critical for gene transcription, so is involved in the assembly and budding of poxviruses [44]. Mitogen-activated protein kinases (MAPKs) are classed into three large subfamilies: JNK, ERK, and MAPK14. The MAPK pathway has a crucial role in viral replication, and previous studies have illustrated that ERK is required for viral replication [45,46,47]. MST1/2, the core component of the Hippo pathway, can inhibit the virus-induced host defense response and the strength of viral replication [35,48]. Growing evidence shows that there are interaction switches between the PI3K/AKT, MAPK, and Hippo signaling pathways, such as Raf. Raf-1 activity is blocked by PI3K/Akt through Akt directly phosphorylating on Ser-259, whereas Raf-1 with phosphorylation on Ser-259 has a dual role in the cross-talk leading to suppressing both the MAPK and Hippo signaling pathways [49,50,51]. According to the above description, we deduced that the MAPK, PI3K/Akt, and Hippo signaling pathways facilitate viral production. In contrast, Raf-1, with cross-talk between these pathways, impedes viral replication. Previous studies illustrated Sorafenib, which is an inhibitor targeting Raf kinases (Raf-1/C-Raf, wide-type B-Raf, and mutant B-Raf) [52], promoted oncoVV-AVL replication in Hela S3 cell lines [24]. Our current data illustrated that oncoVV-AVL replication was significantly downregulated in the presence of the inhibitors of PI3K/Akt, MAPK/ERK, and Hippo/MST pathways. In summary, combined with previous studies, we inferred that the multiple signaling pathways, including PI3K/Akt, Hippo, and MAPK/ERK, may promote oncoVV-AVL replication in Huh7 cells through Raf-1 crosstalk.

Viral infection often changes cellular metabolism to facilitate maximal viral replication. Greseth’s study found that fatty acid synthase inhibitor inhibited the replication and assembly of vaccinia virus [53]. Capsaicin, a component derived from red chili peppers, has significant lipid-lowering activity [54], and can interact with the lipid bilayer [55,56]. Therefore, we tested the effect of capsaicin, and verified that the replications of oncoVV and oncoVV-AVL both decreased. Further studies may explore the details of capsaicin’s effect on oncoVV. Androgen receptor (AR) has been shown to be of relevance for a group of hormone-related diseases, as well as for synthetic metabolic defects such as muscle atrophy and osteoporosis. Moreover, per Tian et al., androgen and AR can stimulate HBV replication in vivo [57]. AR is also closely related to the replication of COVID-19 [58]. Our study also showed that blocking AR by its antagonist inhibited the replication of oncoVV-AVL, while the replication of oncoVV was not affected, implying that expression of AVL by oncoVV may activate the AR-related cell signaling pathway and play a role in viral replication. In summary, our study provides new insight into the anti-HCC activity of oncoVV-AVL, and more comprehensive research on AVL and oncoVV needs to be conducted in the future.

## 4. Materials and Methods

### 4.1. Cell Culture

Human embryonic kidney cell line HEK 293A, and human liver carcinoma cell lines Huh7, Hep-3B, and SK-Hep-1 used in this study were obtained from the Chinese Academy of Sciences. These cells were maintained in DMEM containing 1% penicillin–streptomycin solution and 10% fetal bovine serum, under the condition of 5% CO_2_ at 37 °C.

### 4.2. Cell Viability Assay and Flow Cytometry Determination

Huh7 cells were seeded in 96-well plates. After inoculation for 12 h, the cells were infected with viruses at a multiplicity of infection (MOI) of 5 or MOI of 10. After infection for 24, 48, and 72 h, the cells were harvested for an MTT assay [59]. First, 20 μL thiazolyl blue tetrazolium bromide (MTT) was added to the wells and reacted for 4 h at 37 °C. Then, the liquid in the 96-well plate was completely removed, 150 μL 100% dimethyl sulfoxide (DMSO) was added to every well, and then the absorbance of 570 nm was determined through a microplate reader (Multiskan, Thermo Scientific, Waltham, MA, USA).

To verify cell apoptosis, the virus-infected cell samples were collected and stained with a FITC Annexin V Apoptosis Detection Kit (BD Biosciences, San Jose, CA, USA). Cells were incubated with 1× binding buffer and measured by a flow cytometer (C6, BD Biosciences, San Jose, CA, USA).

### 4.3. Detection of Viral Replication Ability

To detect the viral replication in cells, the Huh7 cells were seeded onto 24-well plates. After 12 h of culture, the cells were infected with oncoVV or oncoVV-AVL. Samples were frozen and thawed three times between −80 °C and room temperature, and viral titers were measured by a TCID_50_ assay using 293A cells in 96-well plates.

To explore how AVL affected oncoVV replication, different agents (Selleck, Houston, TX, USA) were added after cells were maintained for 12 h; 2–3 h later, viruses were added to infect the cells. The experimental steps for testing the viral yields were performed as described above. The agents used in the experiments were U0126 (3 μmol/L), AICAR (750 μmol/L), dorsomorphin (2 μmol/L), XMU-MP-1 (2 μmol/L), KY12420 (4.5 μmol/L), EPI-001 (100 μmol/L), and capsaicin (250 μmol/L).

### 4.4. Western Blot

Cell samples 36 h post-infection were collected, lysed, and quantified. SDS-PAGE electrophoresis was carried out, and samples were electroblotted onto a nitrocellulose membrane. After immersion in 5% skim milk solution for 2 h, the blots were incubated with the primary antibody at 4 °C overnight. Then, the blots were washed and incubated with secondary antibody for 1.5 h. Finally, the protein bands on the membrane were scanned with a chemiluminescence image system. The primary antibodies GAPDH, IRF3, p-IRF3, caspase-3, and caspase-8 were purchased from Cell Signaling Technology, while A27L and OASL were purchased by Abcam and Santa Cruz, respectively. The corresponding secondary antibodies were HRP-conjugated goat anti-rabbit IgG (AS014, ABclonal, Wuhan, China) and HRP-conjugated goat anti-mouse IgG (AS003, ABclonal, Wuhan, China).

### 4.5. qRT-PCR

After cells were infected with the virus for 36 h, RNA was extracted and reverse-transcribed into cDNA with a ReverTra Ace qPCR RT Kit (TOYOBO, Japan). The cDNA was amplified utilizing SYBR^®^ Green Realtime PCR Master Mix (TOYOBO, Osaka, Japan). The expression levels of each gene were normalized by *GAPDH*. The primers used were the same as described previously [20].

### 4.6. Dual Luciferase Reporter Gene Assay

After Huh7 cells were seeded for 12 h, the Renilla luciferase reporter vector PRL-TK and the plasmid with target gene (IRF3, IRF7, AP-1, NF-κB, ISRE) were cotransfected into Huh7 cells with a mass ratio of 1:500 [60]. PRL-TK served as the internal control. Then, 24 h later, Huh7 cells were treated with PBS, oncoVV, or oncoVV-AVL at an MOI of 5 for 36 h. After 36 h of treatment, the luciferase activities were tested by a chemiluminescence apparatus by a Dual-Glo luciferase Assay System (E2920, Promega, Madison, WI, USA).

### 4.7. Transcriptomics Analysis

Huh7 cells were infected with oncoVV-AVL or oncoVV at an MOI of 5 for 36 h. PBS treatment served as the negative control. The cells were collected and submitted to Baygene Biotechnologies Company Limited (Shanghai, China) for transcriptomic sequencing. In brief, Trizol (Invitrogen, Waltham, MA, USA) was used to extract the total RNA, and the assay was conducted with Clariom D human gene chip (Affymetrix, Shanghai, China), GeneChipTM Hybridization, Wash and Stain Kit (Affymetrix, 900720, Shanghai, China), and GeneChipTM WT PLUS Reagent Kit (Affymetrix, 902280, Shanghai, China).

### 4.8. In Vivo Tumor Formation Experiments in Animals

The experimental animals used in this study were 6-week-old female BALB/c nude mice purchased from SLAC Laboratory Animal Company Limited (Shanghai, China). We harvested 4 × 10^6^ Huh7 cells, which we then subcutaneously injected into the abdominal cavity of each mouse. When the tumor volume reached about 200 mm^3^, each mouse in the blank control, negative control, and experimental groups was injected with 100 μL of normal saline, and 1 × 10^7^ PFU oncoVV or oncoVV-AVL, respectively. The volume of tumor was measured and recorded every five days. Tumor volume (V) was calculated as: V = (length × width^2^)/2 [22].

### 4.9. H&E Staining

The tumor issues were removed from the sacrificed tumor-bearing mice, fixed with 4% paraformaldehyde, and stored in 75% ethanol. Paraffin-embedded sectioning and hematoxylin-eosin staining were conducted by HaoKe Biotechnology Company Limited (Hangzhou, China). In brief, the tissues were embedded in paraffin and sectioned away with xylene, then immersed in varying concentrations of ethanol, and washed in distilled water. Subsequently, the tissue sections were stained with hematoxylin and eosin. Finally, the section was dehydrated, fixed, and sealed with a neutral resin. The slides were observed under a light microscope.

### 4.10. Immunohistochemistry Assay

The tumors were removed for immunohistochemistry (IHC) determination at 10 days post viral injection. The IHC assay was conducted by HaoKe Biotechnology Company Limited (Hangzhou, China). In brief, after fixation, the tumor tissue was embedded in paraffin and deparaffinized with xylene and gradient alcohol. Hydrogen peroxide solution was used to remove endogenous catalase, and citrate buffer was used for antigen retrieval. Then, 3% BSA was used for protein blocking. Subsequently, after incubating with primary anti-A27L antibody at 4 °C overnight, the slides were washed with PBS and incubated with secondary HRP-conjugated antibody (ab97051, Abcam, Cambridge, UK) at room temperature for 50 min. Then, the sections were stained with developer, counterstained with hematoxylin, dehydrated, and mounted for inspection.

### 4.11. Statistical Analysis

We used Student’s *t*-test to evaluate whether the differences among different treatment groups were statistically significant, in which *p* < 0.05 or *p* < 0.01 was deemed statistically significant.

## 5. Conclusions

The present study demonstrated that oncoVV-AVL exhibited significant antitumor activity in HCC cells both in vitro and in vivo. Our data revealed that oncoVV-AVL enhanced viral replication through repressing the cell’s antiviral defense response. The signaling pathways, including PI3K/Akt, MAPK/ERK, and Hippo/MST, could strengthen oncoVV-AVL replication in Huh7 cells by cross-talk through Raf-1. Moreover, oncoVV-AVL also engaged in the regulation of cellular metabolism.

## Data Availability

Data are contained within the article.
